# Effects of acute footbath before and after glucose ingestion on arterial stiffness

**DOI:** 10.3164/jcbn.18-71

**Published:** 2019-01-16

**Authors:** Ryota Kobayashi, Yuto Hashimoto, Takanobu Okamoto

**Affiliations:** 1 Center for Fundamental Education, Teikyo University of Science, 2-2-1 Senju, Sakuragi, Adachi-ku, Tokyo 120-0045, Japan; 2 Graduate School of Health and Sport Science, Nippon Sport Science University, 7-1-1 Fukasawa, Setagaya-ku, Tokyo 158-8508, Japan; 3 Department of Exercise Physiology, Nippon Sport Science University, 7-1-1 Fukasawa, Setagaya-ku, Tokyo 158-8508, Japan

**Keywords:** footbath, glucose ingestion, leg arterial stiffness, ankle blood pressure

## Abstract

The present study investigated the acute effect of a footbath on increases in arterial stiffness after glucose ingestion in healthy young women. Nine healthy young women (aged 18.4 ± 0.2 years; mean ± SE) completed three trials in random order. They started a footbath before 75-g oral glucose ingestion, a footbath after 75-g oral glucose ingestion and no footbath (control) trials. Aortic (carotid–femoral) and leg (femoral–ankle) pulse wave velocity, the carotid augmentation index, carotid, brachial and ankle blood pressure, heart rate, blood glucose levels, insulin levels and sublingual temperature were measured before (baseline) and at 15, 30, 60 and 90 min after the 75-g oral glucose ingestion. Aortic pulse wave velocity and brachial systolic blood pressure did not change from baseline to after the 75-g oral glucose ingestion in all trials. Leg pulse wave velocity and ankle systolic blood pressure were increased from baseline to after the 75-g oral glucose ingestion in the footbath after glucose ingestion and control trials, but not in the footbath before glucose ingestion trial. These results suggest that a footbath effectively suppresses the increase in leg arterial stiffness after glucose ingestion when implemented before glucose ingestion.

## Introduction

Increased central (aortic) and peripheral (leg) arterial stiffness is an important determinant of cardiovascular risk.^(1)^ Moreover, increased ankle systolic blood pressure (BP) has been proposed as an independent risk factor for development of cardiovascular disease.^(2)^ Wohlfahrt *et al.*^(3)^ reported that leg arterial stiffness is correlated with ankle systolic BP. Therefore, inhibiting an increase in leg arterial stiffness and ankle systolic BP might improve health.

Arterial stiffness acutely increases after glucose ingestion.^(4)^ The 75-g oral glucose tolerance test is used as a model of high-carbohydrate meals to investigate the effects of post-prandial hyperglycemia on arterial stiffness.^(5)^ Our previous studies showed that leg arterial stiffness and ankle systolic BP acutely increased after the 75-g oral glucose ingestion and leg pulse wave velocity (PWV) and ankle systolic BP positively correlated at 30 min after the 75-g oral glucose ingestion.^(5)^ An increased cardiovascular risk due to post-prandial hyperglycemia associated with post-prandial impaired arterial function, such as arterial stiffening.^(6)^ Therefore, acute increases in leg arterial stiffness after glucose ingestion should be inhibited.

Thermal therapy decreases arterial stiffness in humans. Zhu *et al.*^(7)^ found that systemic arterial stiffness decreases after an acute warm shower in healthy young humans. Footbaths are a beloved tradition in most Japanese people and they have become popular because they are easy to carry out without undressing. Compared with systemic thermal therapy, a footbath exposes only part of the body to water, which is more convenient and accessible in daily life. Currently, a footbath can be easily carried out in parks, restaurants, and cafes. Hu *et al.*^(8)^ showed that a footbath (41–43°C) for 30 min decreased systemic arterial stiffness after 0 min in healthy young women. Additionally, Kosaki *et al.*^(9)^ found that leg arterial stiffness was decreased after a footbath in healthy young men. Therefore, an acute footbath might suppress an increase in leg arterial stiffness after glucose ingestion (i.e., high-carbohydrate meals). However, the optimal timing to perform a footbath in terms of suppressing an increase in leg arterial stiffness after glucose ingestion is unknown.

The present study aimed to investigate the acute effect of a footbath on arterial stiffness after glucose ingestion in young women. We hypothesized that an increase in arterial stiffness after glucose ingestion is suppressed by an acute footbath before, rather than after, glucose ingestion.

## Materials and Methods

### Subjects

Nine healthy young women (age, 18.4 ± 0.2 years; height, 158.4 ± 1.2 cm; weight, 53.8 ± 2.8 kg; mean ± SE) participated in the present study. All of these women lived a sedentary lifestyle (≥2 years without regular exercise as assessed by the international physical activity questionnaire) and were normotensive (<120/80 mmHg) non-smokers without symptoms or a history of any overt chronic disease. All of the participants were studied during the early follicular phase of the menstrual cycle to avoid any hormonal influences on arterial stiffness. None of the female participants was taking oral contraceptives (Table 1). All of the participants were fully informed about the experimental procedures, as well as the purpose of the study before providing written, informed consent to participate. The ethics committee at Nippon Sport Science University approved this study, which proceeded in accordance with the guidelines for human experimentation published by our institutional review board. This study also conformed to the principles of the Declaration of Helsinki.

### Study design and Sample size

**Fig. 1:**
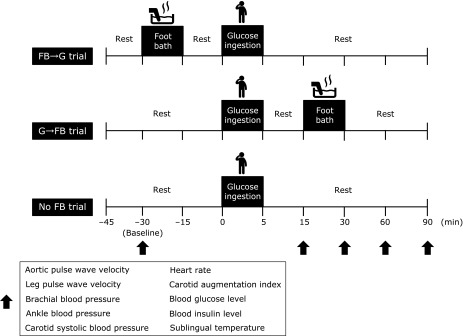
Study design. Aortic and leg PWV, carotid AIx, brachial, ankle and carotid BP, HR, BG and insulin levels and sublingual temperature were measured before (baseline, −30 min) and at 15, 30, 60 and 90 min after the 75-g oral glucose ingestion. PWV, pulse wave velocity; AIx, augmentation index; BP, blood pressure; HR, heart rate; BG, blood glucose; FB→G trial, footbath at 30 min before the 75-g oral glucose ingestion trial; G→FB trial, footbath at 15 min after the 75-g oral glucose ingestion trial; No FB trial, no footbath before and after the 75-g oral glucose ingestion trial.

Each participant completed three trials in random order as follows: a footbath at 30 min before the 75-g oral glucose ingestion (FB→G trial), a footbath at 15 min after the 75-g oral glucose ingestion (G→FB trial), and no footbath before and after the 75-g oral glucose ingestion (No FB trial) trials. First, participants were asked to lie still for at least 15 min in a temperature-controlled room (approximately 24°C) before baseline measurements were obtained. During the interventions, the subjects were then asked to rest still in a comfortable chair and put their lower legs and feet into a bathtub (diameter: 42 cm, depth: 40 cm) containing hot water (approximately 43°C, approximately 40 cm of hot water). To maintain the temperature of the water in the bathtub at 43°C for 15 min, hot water (43°C) was continually added to the bathtub during the 15 min trial using a hot water supply system (BC60V3; Rinnai Corporation, Nagoya, Japan). All of the participants abstained from alcohol, caffeinated beverages and intense physical activity (exercise) for 24 h before being tested and reported to the laboratory after fasting for 10–12 h. They rested for 15 min in the supine position and then aortic and leg arterial stiffness, carotid, brachial, and ankle BP, heart rate (HR), the carotid augmentation index (AIx), BG levels, insulin levels and sublingual temperature were measured at 30 min before (baseline), and at 15, 30, 60 and 90 min after the 75-g glucose ingestion (Fig. 1). Before starting the study, we performed power analyses using G*Power 3 to determine the appropriate sample size a sample of 9 females was needed.

### Body composition

Body composition was determined by bioelectric impedance using an InBody770 body composition analyzer (Biospace Co. Ltd., Seoul, Korea).

### Pulse wave velocity

PWV was measured as follows. Briefly, carotid–femoral and femoral–ankle arterial pulse waves were obtained in triplicate using an arterial applanation tonometer that incorporated an array of 15 transducers. Carotid–femoral (cf) and femoral–ankle (fa) PWV reflect aortic and leg arterial stiffness, respectively.^(11)^ Electrocardiography was then performed, and bilateral brachial and ankle BP, and carotid and femoral arterial pulse waves were simultaneously measured using a form PWV/ABI vascular testing device (Omron-Colin Co. Ltd., Kyoto, Japan). Carotid and femoral arterial pressure waveforms were stored for 30 s via applanation tonometry sensors that were attached to the left common carotid and left common femoral arteries. We calculated PWV from the distance between two arterial recording sites divided by the transit time, and pulse wave transit time was determined from the interval between proximal and distal “foot” waveforms. Femoral–ankle PWV was obtained as the transit time between the femoral artery (tonometer) and ankle sites. Intervals between the right brachial and post-tibial arteries, between the carotid and femoral arteries (Tcf), and between the femoral and post-tibial arteries were determined. The path length from the carotid to the femoral artery (Dcf) was assessed in duplicate with a random zero length measurement over the surface of the body using a non-elastic tape measure. Aortic PWV was calculated as Dcf/Tcf. The carotid AIx, a proposed indicator of the magnitude of wave reflections, was obtained from pressure waveforms.^(11)^ The daily coefficients of variation (CVs) at the laboratory were 3 ± 1, 3 ± 2 and 6 ± 1% for carotid–femoral PWV, femoral–ankle PWV, and the carotid AIx, respectively.

### Blood pressure and heart rate

Brachial and ankle systolic BP (SBP), mean BP (MBP), diastolic BP (DBP), pulse pressure and HR while resting in the supine position were measured in triplicate using an automated oscillometric form PWV/ABI device (Omron-Colin Co. Ltd.) over the brachial and ankle arteries. Carotid SBP, which has been proposed as an indicator of the magnitude of wave reflections, was also obtained from the pressure waveforms.^(11)^

### Blood glucose and insulin levels

Venous blood was withdrawn from participants via a catheter in the right or left arm at 30 min before and at 15, 30, 60 and 90 min after glucose ingestion. Levels of BG and insulin were assayed using the UV-hexokinase method and a CLEIA, respectively.

### 75-g oral glucose ingestion

We used TrelanG75 (Ajinomoto Pharma. Co. Ltd., Tokyo, Japan) in the morning after an overnight (10–12 h) fast for 75-g glucose ingestion. The glucose beverage (225 ml) was within the standard for adult humans and was consumed within 5 min.^(12)^

### Sublingual temperature

Sublingual temperature was measured as an estimate of core temperature. In this study, we measured the subject’s sublingual temperature by inserting the probe of a Terumo C531 electrical thermometer (Terumo Co. Ltd., Tokyo, Japan) into the sublingual of the subjects. At each time point, the temperature was measured twice. The two results were averaged for later analysis. There were no cases of greater than a 5% difference between the readings.

### Statistical analysis

All data are presented as mean ± SE. Data were analyzed using two-way repeated-measures ANOVA (trial-by-time). Significant differences among mean values were identified using the Bonferroni post hoc test. Data were statistically analyzed using SPSS ver. 22 (IBM, Armonk, NY). Statistical significance was set at *p*<0.05.

## Results

### Arterial stiffness

**Fig. 2:**
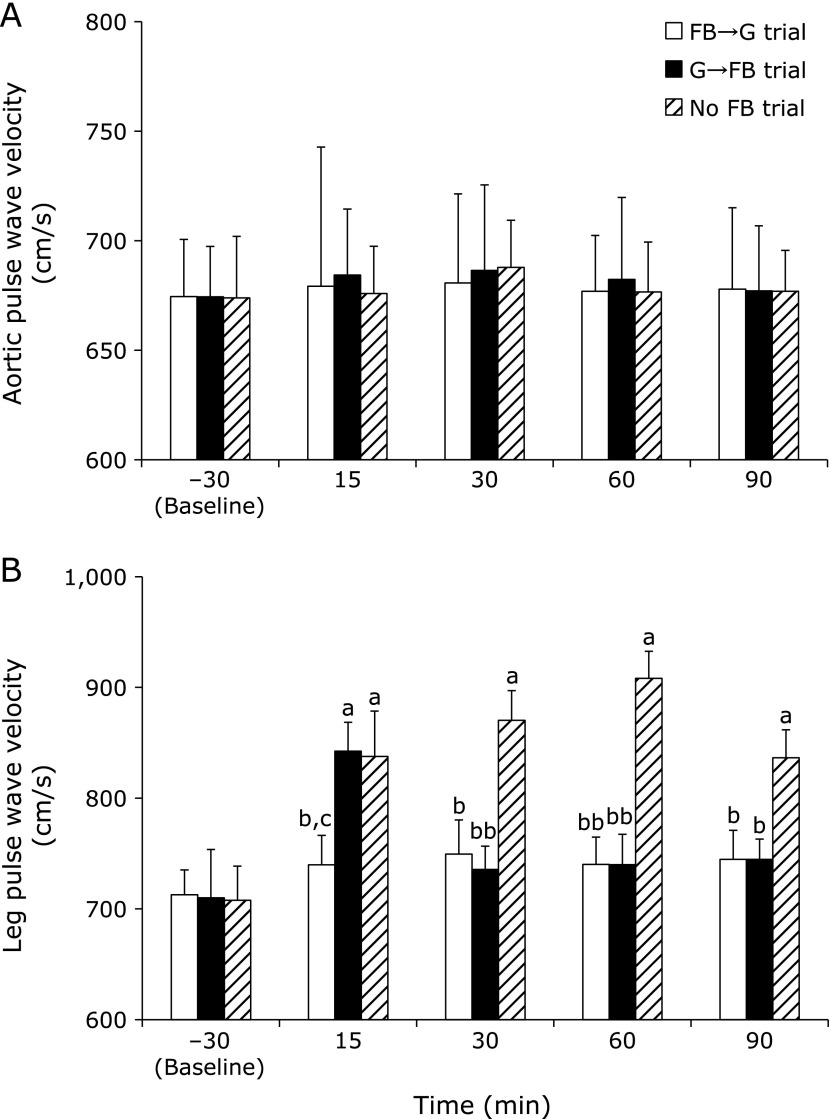
Change in aortic PWV (A) and leg PWV (B) before (baseline, −30 min) at 15, 30, 60 and 90 min after the 75-g oral glucose ingestion in all trials. Values are mean ± SE. PWV, pulse wave velocity; FB→G trial, footbath at 30 min before the 75-g oral glucose ingestion trial; G→FB trial, footbath at 15 min after the 75-g oral glucose ingestion trial; No FB trial, no footbath before and after the 75-g oral glucose ingestion trial. ^a^*p*<0.05 vs baseline. ^b^*p*<0.05 and ^bb^*p*<0.01 vs No FB trial, ^c^*p*<0.05 vs G→FB trial.

Aortic PWV did not change from baseline to 15, 30, 60 and 90 min after the 75-g glucose ingestion in all trials and did not differ between them (Fig. 2A). Leg PWV was significantly higher in the No FB trial compared with the FB→G trial at 15 (*p*<0.05), 30 (*p*<0.05), 60 (*p*<0.01) and 90 (*p*<0.05) min after the 75-g oral glucose ingestion. Leg PWV was significantly higher in the No FB trial compared with the G→FB trial at 30 (*p*<0.01), 60 (*p*<0.01) and 90 (*p*<0.05) min after the 75-g oral glucose ingestion. Leg PWV was significantly higher in the G→FB trial compared with the FB→G trial at 15 min after the 75-g oral glucose ingestion (*p*<0.05). In the No FB trial, leg PWV was significantly higher at 15, 30, 60 and 90 min compared with baseline (*p*<0.05). In the FB→G trial, leg PWV did not change after the 75-g oral glucose ingestion. In the G→FB trial, leg PWV was significantly higher at 15 min compared with baseline (*p*<0.05) and did not change at 30, 60 and 90 min compared with baseline (Fig. 2B).

### Brachial blood pressure

After glucose ingestion, brachial SBP, MBP and DBP did not change in the all trials (Table 2).

### Ankle and carotid blood pressure, carotid AIx, heart rate and sublingual temperature

After glucose ingestion, ankle SBP, MBP and DBP were significantly increased in the No FB and G→FB trials (*p*<0.05), but did not change in the FB→G trial. Ankle SBP, MBP and DBP were significantly lower in the FB→G trial compared with the No FB and G→FB trials at 15 min (*p*<0.05) and lower in the FB→G trial compared with the No FB trial at 30 and 60 min (*p*<0.05). Carotid SBP, carotid AIx, heart rate and sublingual temperature did not change from baseline to 15, 30, 60 and 90 min after glucose ingestion in all trials and did not differ between them (Table 3).

### Blood glucose and insulin levels

BG level was significantly higher in the G→FB trial compared with the FB→G trial at 15 (*p*<0.05) min after the 75-g oral glucose ingestion. BG level was significantly higher in the No FB compared with the FB→G trial at 15 (*p*<0.05), 30 (*p*<0.05) and 60 (*p*<0.05) min after the 75-g oral glucose ingestion. In the No FB trial, glucose level was significantly higher at 15 (*p*<0.01), 30 (*p*<0.01), 60 (*p*<0.01) and 90 (*p*<0.05) min compared with baseline. In the FB→G trial, glucose level was significantly higher at 15 (*p*<0.01) min compared with baseline. In the G→FB trial, glucose level was significantly higher at 15 (*p*<0.01), 30 (*p*<0.05) and 60 (*p*<0.05) min compared with baseline. Insulin level was significantly higher in the G→FB trial compared with the FB→G trial at 15 (*p*<0.05) min after the 75-g oral glucose ingestion. Insulin level was significantly higher in the No FB trial compared with the FB→G trial at 15 (*p*<0.05), 30 (*p*<0.05), 60 (*p*<0.05) and 90 (*p*<0.05) min after the 75-g oral glucose ingestion. In the No FB trial, insulin level was significantly higher at 15, 30, 60 and 90 min compared with baseline (*p*<0.01). In the FB→G trial, insulin level was significantly higher at 15 min compared with baseline (*p*<0.01). In the G→FB trial, insulin level was significantly higher at 15 (*p*<0.01) and 30 (*p*<0.01) min compared with baseline (Table 4).

## Discussion

The main novel finding of this study was that the leg arterial stiffness was increased after the 75-g glucose ingestion compared with baseline in the No FB and G→FB trials, but not in the FB→G trial. These results suggest that a footbath effectively suppresses the increase in peripheral arterial stiffness after glucose ingestion when implemented before glucose ingestion.

Arterial stiffness acutely increases with high carbohydrate meals.^(13)^ Grassi *et al.*^(4)^ and Gomez-Sanchez *et al.*^(14)^ reported that systemic and peripheral arterial stiffness increases after glucose ingestion in healthy volunteers. We previously reported that leg arterial stiffness was acutely increased after the 75-g glucose ingestion.^(15)^ The current study showed that leg arterial stiffness was increased at 15, 30, 60 and 90 min after the 75-g glucose ingestion in the No FB and G→FB trials, whereas aortic arterial stiffness did not change after the 75-g oral glucose test in all trials. We previously reported that aortic arterial stiffness does not change in healthy young people after glucose ingestion.^(15)^ Therefore, aortic arterial stiffness after glucose ingestion might not change in young people.

Thermal therapy decreases arterial stiffness in humans. Zhu *et al.*^(16)^ showed that systemic arterial stiffness was significantly decreased after a 20 min warm shower and remained depressed for at least 30 min in healthy young men. Localized thermal therapy, such as a footbath, is practical and accessible in daily life. Hu *et al.*^(8)^ reported that a footbath with both legs induced a decrease in systemic arterial stiffness at 0 min after a footbath compared with before a footbath in healthy young women. Kosaki *et al.*^(9)^ reported that leg arterial stiffness was decreased at 30 min after heat treatment of the lower leg (temperature of approximately 43°C) in healthy young men. Our study showed that leg arterial stiffness was increased after the 75-g glucose ingestion compared with baseline in the No FB and G→FB trials, but not in the FB→G trial. Therefore, a footbath before glucose ingestion (i.e., high-carbohydrate meals) might be the optimal timing to suppress an increase in arterial stiffness after glucose ingestion (i.e., high-carbohydrate meals). Consequently, an acute footbath before glucose ingestion might benefit cardiovascular health. In addition, increased postoperative arterial stiffness (a risk factor for cardiovascular disease) can be suppressed by pre-meal acute aerobic exercise, but has a high physical load. On the other hand, anyone can do the footbath. That is, footbath has clinical significance as a method to suppress increase in arterial stiffness after meals (That is, bathing → meal).

The present study was not designed to examine possible mechanisms through which a footbath at 30 min before glucose ingestion might be the optimal timing to suppress an increase in arterial stiffness after glucose ingestion. However, we propose the following explanations for this finding. An increase in leg arterial stiffness after glucose ingestion might be due to increased ankle SBP after glucose ingestion. In fact, our previous study showed a positive correlation between leg arterial stiffness and ankle SBP after the 75-g glucose ingestion.^(5)^ In the current study, leg arterial stiffness and ankle SBP were increased after the 75-g glucose ingestion compared with baseline in the No FB and G→FB trials, but did not change in the FB→G trial. Therefore, a footbath before glucose ingestion suppressing an increase in leg arterial stiffness might be a mechanism associated with suppressing an increase in ankle SBP. With regard to another mechanism, endothelial cells play an important role in regulating vascular activity by producing vasoactive substances, such as nitric oxide, which participates in regulation of arterial stiffness.^(17,18)^ Endothelial function acutely decreases after glucose ingestion.^(4)^ Furthermore, a previous study showed that raising the core temperature to 42°C for 15 min in rats resulted in a significant increase in endothelial nitric oxide synthase activity.^(19)^ A footbath may increase endothelial nitric oxide synthase activity, resulting in increased blood flow and shear stress to modulate arterial stiffness.^(20)^ Green *et al.*^(21)^ reported that improvement in endothelial function was related to an increase in shear stress associated with elevated blood flow during heating. Arterial stiffness and MBP are influenced by vascular endothelial function, which decreases after glucose ingestion.^(22–24)^ Changes in arterial stiffness are influenced by MBP.^(25)^ Our study showed that leg arterial stiffness and MBP were increased at 30 and 60 min after the 75-g glucose ingestion in the No FB trial, whereas leg arterial stiffness and MBP did not change after the 75-g glucose ingestion in FB→G trial. SBP decreases due to an increase in nitric oxide.^(26)^ Therefore, suppression of increased leg arterial stiffness after glucose ingestion due to a footbath before ingestion might be associated with suppresses increases SBP by change in peripheral endothelial function (change in nitric oxide) after glucose ingestion. However, we did not measure peripheral endothelial function, which is a limitation of this study. To date, few studies have investigated the effects of heat treatment on BG and insulin levels after glucose ingestion. Our study showed that BG and insulin levels were increased in all trials after the 75-g glucose ingestion, BG and insulin levels after the 75-g oral glucose ingestion was significantly higher in the G→FB and No FB trials compared with the FB→G trial. Therefore, acute hyperthermia treatment before glucose ingestion might be able to suppress increases in BG levels after glucose ingestion. Hooper *et al.*^(27)^ found that chronic hyperthermia treatment improved BG levels. Therefore, the effect of repetitive thermal stimulation on blood glucose levels after ingestion of glucose should be investigated.

Arterial stiffness increases in healthy individuals after glucose-based snacks (white chocolate bars, 100 g).^(28)^ Therefore, further studies are required to determine when acute footbath should occur to maximally affect arterial stiffness after consuming standard forms of glucose.

The present study has several limitations. Our participants were healthy young women, which precludes generalizing our findings to middle-aged, older, or male individuals. Moreover, the sample size was small (*n* = 9), although the magnitude of the effect on arterial stiffness was adequate and similar to that in previous studies of acute aerobic exercise on arterial stiffness after meals.^(28)^ Additionally, peripheral endothelial function, oxidative stress, and sympathetic nervous system activity, which could have important effects on arterial stiffness, were not assessed.

In conclusions, the leg arterial stiffness was increased after the 75-g glucose ingestion compared with baseline in the No FB and G→FB trials, but not in the FB→G trial. These results suggest that the most effective time for a footbath to suppress an increase in leg arterial stiffness after glucose ingestion (i.e., high-carbohydrate meals) by healthy, young, adult females is before glucose ingestion.

## Figures and Tables

**Table 1:** Subject characteristics (*n* = 9)

Variable	Value
Age (years)	18.4 ± 0.2
Height (cm)	158.4 ± 1.2
Weight (kg)	53.8 ± 2.8
Body fat (%)	28.4 ± 1.2
BMI (kg/m^2^)	21.3 ± 1.0
LBM (kg)	39.7 ± 2.0

Values are mean ± SE. BMI, body mass index; LBM, lean body mass.

**Table 2:** Changes in brachial SBP, MBP and DBP before (baseline, –30 min) and after glucose ingestion in all trials

Variable	Trial	Baseline (−30 min)	15 min	30 min	60 min	90 min
Brachial SBP (mmHg)	FB→G trial	100.1 ± 2.1	101.1 ± 1.0	103.9 ± 1.9	102.0 ± 2.3	102.0 ± 3.0
	G→FB trial	101.9 ± 3.3	101.4 ± 1.7	101.2 ± 1.1	102.2 ± 0.9	101.1 ± 2.5
	No FB trial	100.1 ± 1.9	101.3 ± 1.9	101.2 ± 0.6	100.8 ± 1.6	101.7 ± 1.1
Brachial MBP (mmHg)	FB→G trial	72.1 ± 2.1	73.0 ± 1.5	73.9 ± 1.5	73.0 ± 1.6	72.6 ± 1.5
	G→FB trial	73.0 ± 2.3	74.9 ± 2.6	75.0 ± 2.2	73.5 ± 1.6	72.9 ± 1.6
	No FB trial	72.9 ± 2.4	74.6 ± 2.0	75.8 ± 1.8	73.0 ± 1.6	74.3 ± 1.3
Brachial DBP (mmHg)	FB→G trial	58.1 ± 2.4	58.9 ± 2.0	58.9 ± 1.6	58.1 ± 1.7	57.9 ± 1.1
	G→FB trial	58.5 ± 2.1	59.6 ± 2.2	59.7 ± 1.6	59.1 ± 1.6	58.3 ± 1.1
	No FB trial	58.3 ± 2.4	59.3 ± 1.9	60.1 ± 1.7	58.2 ± 1.5	58.8 ± 1.2

Values are mean ± SE. SBP, systolic blood pressure; MBP, mean blood pressure; DBP, diastolic blood pressure; FB→G trial, footbath at 30 min before the 75-g oral glucose ingestion trial; G→FB trial, footbath at 15 min after the 75-g oral glucose ingestion trial; No FB trial, no footbath before and after the 75-g oral glucose ingestion trial.

**Table 3:** Changes in ankle SBP, MBP, DBP, carotid SBP, HR, carotid AIx and sublingual temperature before (baseline, −30 min) and after glucose ingestion in all trials

Variable	trial	Baseline (−30 min)	15 min	30 min	60 min	90 min
Ankle SBP (mmHg)	FB→G trial	110.6 ± 2.2	108.2 ± 2.8^d,e^	111.1 ± 2.1^d^	111.1 ± 2.3^d^	111.3 ± 1.5^dd^
	G→FB trial	110.3 ± 4.2	120.3 ± 2.4^aa^	113.0 ± 2.7^b^	114.7 ± 3.3	114.6 ± 1.7^d^
	No FB trial	110.8 ± 3.1	120.5 ± 3.3^aa^	121.0 ± 2.5^aa^	121.6 ± 2.3^aa^	121.6 ± 1.6^a^
Ankle MBP (mmHg)	FB→G trial	73.5 ± 1.8	72.2 ± 2.1^d,ee^	74.5 ± 1.6^d^	74.1 ± 1.5^d^	74.3 ± 1.0
	G→FB trial	73.7 ± 2.8	81.6 ± 1.9^aa^	76.7 ± 1.7	76.7 ± 2.1^b^	78.1 ± 1.9
	No FB trial	73.2 ± 1.8	81.2 ± 2.0^aa^	82.9 ± 2.4^aa^	79.7 ± 1.6^aa^	75.6 ± 2.8^cc^
Ankle DBP (mmHg)	FB→G trial	54.9 ± 1.7	54.3 ± 1.9^d,e^	56.1 ± 1.5^d^	55.6 ± 1.2^d^	55.9 ± 1.3
	G→FB trial	55.4 ± 2.4	62.2 ± 1.9^aa^	58.5 ± 1.7	57.6 ± 1.7^b^	58.3 ± 1.7
	No FB trial	54.5 ± 1.7	61.5 ± 1.9^aa^	62.5 ± 1.8^aa^	61.1 ± 1.4^aa^	56.3 ± 2.7^cc^
Carotid SBP (mmHg)	FB→G trial	113.3 ± 2.6	114.1 ± 2.2	114.9 ± 2.4	112.9 ± 4.0	112.3 ± 3.4
	G→FB trial	113.0 ± 4.3	115.3 ± 3.4	115.6 ± 3.2	113.8 ± 2.7	113.2 ± 5.8
	No FB trial	113.4 ± 2.9	115.8 ± 3.2	116.9 ± 4.5	114.3 ± 5.5	113.4 ± 2.5
HR (beats/min)	FB→G trial	58.2 ± 2.2	62.0 ± 2.2	63.8 ± 2.5	61.9 ± 2.3	61.9 ± 2.0
	G→FB trial	58.1 ± 2.6	61.9 ± 2.7	63.7 ± 3.5	61.8 ± 2.4	61.9 ± 2.4
	No FB trial	58.0 ± 2.1	62.4 ± 3.0	63.9 ± 2.3	62.1 ± 2.9	62.0 ± 2.8
Carotid AIx (%)	FB→G trial	−15.8 ± 3.6	−20.0 ± 3.0	−20.5 ± 3.4	−19.2 ± 3.2	−15.9 ± 4.1
	G→FB trial	−15.6 ± 3.8	–17.8 ± 4.4	−22.2 ± 4.4	−14.2 ± 3.4	−15.0 ± 4.2
	No FB trial	−15.7 ± 3.7	−19.8 ± 4.8	−26.5 ± 5.5	−21.3 ± 3.7	−16.9 ± 3.1
Sublingual temperature (°C)	FB→G trial	36.7 ± 0.1	36.6 ± 0.1	36.6 ± 0.1	36.4 ± 0.1	36.3 ± 0.1
	G→FB trial	36.7 ± 0.1	36.4 ± 0.1	36.6 ± 0.1	36.4 ± 0.1	36.3 ± 0.2
	No FB trial	36.8 ± 0.1	36.4 ± 0.1	36.6 ± 0.1	36.4 ± 0.1	36.4 ± 0.2

Values are mean ± SE. SBP, systolic blood pressure; MBP, mean blood pressure; DBP, diastolic blood pressure; HR, heart rate; AIx, augmentation index; FB→G trial, footbath at 30 min before the 75-g oral glucose ingestion trial; G→FB trial, footbath at 15 min after the 75-g oral glucose ingestion trial; No FB trial, no footbath before and after the 75-g oral glucose ingestion trial. ^a^*p*<0.05 and ^aa^*p*<0.01 vs baseline. ^b^*p*<0.05 and ^bb^*p*<0.01 vs 15 min. ^cc^*p*<0.01 vs 30 min. ^d^*p*<0.05 and ^dd^*p*<0.01 vs No FB trial. ^e^*p*<0.05 and ^ee^*p*<0.01 vs G→FB trial.

**Table 4:** Changes in BG and insulin levels before (baseline, −30 min) and after glucose ingestion in all trials

Variable	Trial	Baseline (−30 min)	15 min	30 min	60 min	90 min
BG level (mg/dl)	FB→G trial	86.8 ± 5.5	123.0 ± 6.5^aa^	122.6 ± 7.2	121.7 ± 8.0	116.8 ± 9.8
	G→FB trial	83.4 ± 1.9	132.9 ± 5.3^aa,b^	127.0 ± 9.0^a^	122.7 ± 8.3^a^	117.3 ± 5.8
	No FB trial	82.3 ± 2.3	132.8 ± 5.8^aa,b^	139.2 ± 15.3^aa,b^	141.9 ± 15.1^aa^^,^^b^	120.8 ± 15.2^a^
Insulin level (µIU/ml)	FB→G trial	5.4 ± 0.9	35.7 ± 6.7^aa^	36.2 ± 6.0^b^	33.4 ± 4.4	32.5 ± 4.0
	G→FB trial	5.5 ± 0.6	40.7 ± 5.4^aa^^,^^b^	35.0 ± 5.4^aa^	33.1 ± 5.1	31.9 ± 3.7
	No FB trial	4.6 ± 0.8	44.7 ± 4.1^aa^^,^^b^	52.2 ± 10.0^aa^^,^^b^	52.3 ± 14.2^aa^^,^^b^	46.0 ± 14.0^aa^^,^^b^

Values are mean ± SE. BG, blood glucose; FB→G trial, footbath at 30 min before the 75-g oral glucose ingestion trial; G→FB trial, footbath at 15 min after the 75-g oral glucose ingestion trial; No FB trial, no footbath before and after the 75-g oral glucose ingestion trial. ^a^*p*<0.05 and ^aa^*p*<0.01 vs baseline. ^b^*p*<0.05 vs FB→G trial.
